# Lectures on Osteology, Including the Ligaments

**Published:** 1845-01

**Authors:** 


### Lectures on Osteology, including the Ligaments which connect the Bones of the Human Skeleton. By *B. B. Cooper*, F.R.S. Highley, London, 1844. 8vo. pp. 270.

This work, a second and improved edition of Mr. Bransby Cooper's first volume of "Lectures on Anatomy," furnishes a very good description of the various bones and joints of the human skeleton; the different accidents to which each part is liable, and the most appropriate means of relief, being concisely stated in smaller type at the foot of each article. Practitioners of medicine forming the great bulk of our readers, the noticing a production intended as a class-book for students may seem somewhat out of place: but we do so for two reasons. In the first place, a young man naturally consults his seniors in the profession as to what books he had better purchase; and, seeing the tempting variety offered to his notice, and the limited means frequently at his disposal, we think he only acts prudently in so doing. But, secondly, and chiefly, because we think this is one of a class of books a practitioner should consider himself responsible for all pupils committed to his care, being familiar with the contents of prior to the expiration of their pupillage, and the formal commencement of their studies at the schools. This is a very important matter, but we believe the discussion of it comes almost too late. The days of the appenticeship system are numbered. But why? Not so much from any inherent rottenness in the thing itself, as from the neglect of those who have had the working of it out, in not having allowed it to participate in the spirit of modern improvement. Formerly, when the apothecary was the mere humble attendant upon the physician, and when the careful preparation of the complex forms of prescription then in vogue, constituted the great object of his existence, an apprenticeship passed in culling simples, and in pestle and mortar slavery, seemed not very unreasonable, especially as the act of dispensing was necessarily a more tedious and difficult matter than at present. But, after the apothecary had succeeded in elevating his position in the profession to that rank which the general practitioner now occupies, it became a gross and palpable inconsistency on his part still to limit the instruction of his pupils to mere pharmaceutical matters, and to neglect preparing them for that wider sphere of activity and usefulness which had been opened out to them. It is a subject of daily complaint that the curricula of the examining bodies compress so large a quantity and great a variety of requirements within so short a space of time. They, however, have no option if they wish to maintain the present high standard of professional education, and yet not preclude young men of comparatively limited means from endeavouring to attain it. The only mode of diminishing the inconvenience is for students to commence their attendance upon the various lectures in a better state of preparation than they do at present, and which it is impossible for them to do, as long as the mere compounding of medicines forms the chief employment of their apprenticeship. Under any arrangement much must remain to be done in London, for which the time allotted is barely sufficient; but when this time is trenched upon by commencing learning that which ought already to have been acquired, the case often becomes desperate, the bewildered student, harassed and frightened by the multitude of objects which are demanding his attention, throws himself into the arms of the *grinder* as his deliverer, and spends hour after hour in repulsive, mechanical, parrot-like labour, disgusting himself with studies, which, judiciously conducted, possess unequalled attractions, and losing the opportunities which his residence in a large capital can alone afford him, of observing examples of disease under every possible variety of circumstance.

During his apprenticeship the pupil should have the facilities offered him of obtaining a general idea of the structure and functions of the frame, and a complete knowledge of the bony portions of it. Of Chemistry, Materia Medica, Natural Philosophy, and Botany, by the aid of some of the excellent works now published, he might also acquire all the outlines. Thus informed, he arrives at the schools fit and ready to make the most of his opportunities; and when there, Practical Anatomy and Chemistry, and the minute and constant Observation of the Sick, become to him improving and interesting occupations, and should indeed chiefly absorb his attention. Many practitioners, we are aware, afford these facilities to their pupils; but that the great bulk do not, the very ignorant condition which many young men arrive in, and the consequent loss of time they incur, is a sufficient proof. Did all do so there could be no solid objection to apprenticeships; for no one wishes young men to be empowered to enter the profession at an earlier age than they do at present; and where could they so well pass the three or four years intervening between their leaving school and commencing attendance in the large towns, as under the careful inspection of one already versed in the knowledge they seek to attain, and willing to afford them assistance and instruction?

To revert to Mr. Cooper's book: we have only to state that the descriptions of the various bones and joints are very complete; while the surgical observations appended are sufficiently so to point out to the student the practical bearings of the subject—thus at once showing to him the importance of the study he is engaged in, and relieving, in some degree, the tedium of its pursuit. One or two of these we may extract.

"*Treatment of Fractures.*—Immediately after fracture of any bone, when there is a tendency to great tumefaction, it is wrong to apply either splints or bandage, for any restriction of swelling is liable to produce gangrene: under these circumstances, the limb should be placed on a pillow in a semi-flexed position, so that the muscles may be perfectly relaxed, and the bones placed as nearly as possible in their natural position: which circumstances may be ascertained, whatever may be the swelling, by the immediate comparative ease of the patient. An evaporating lotion is then to be used; or should there be any tendency to involuntary contraction of the muscles, strips of soap plaster may be gently applied around the limb, which, by causing a secretion beneath it, diminishes the irritability of the muscles, as well as the urgency of the inflammation. If it can be avoided, purgative medicines should not be given, as they would produce a necessity for frequent change of position: but should their use be considered essential, such medicines are to be given as are least likely to keep up a continual action on the bowels. As soon as the tumefaction and inflammation have subsided, which, under the treatment recommended, generally happens in three or four days, well-padded splints should be applied, and retained in their situation by broad pieces of tape resting firmly on the splints, but which should not be anywhere in contact with the limb.

"It has been stated, that a bandage should never be applied immediately after the occurrence of fracture: however, it may be considered as an exception to this rule, that when a portion of fractured bone has wounded and irritated a muscle, a bandage is the best means of relieving its spasmodic action. When fracture of a bone happens in the neighbourhood of, or passes into a joint, local bleeding, by means of leeches, is always necessary, and may require to be frequently repeated: even the necessity for general bleeding may sometimes be indicated, when there is much constitutional irritation: in which case, calomel and opium will also be found of the greatest service. In fractures into joints, when inflammation becomes so violent that the surgeon sees that anchylosis must necessarily occur, the joint should be placed in such a position, as to render the limb as useful as possible. Under these circumstances, for instance, if the elbow-joint be the one affected, the fore-arm should be semi-flexed; by which position the patient will afterwards be able to feed himself. In the knee-joint, the leg should be very slightly flexed upon the thigh; by which method he is better able to direct the foot, and the limb is rendered more manageable in the sitting posture. In the ancle-joint, we should endeavour to procure a union with the foot perfectly flat; whereby the patient will afterwards enjoy very considerable use of his limb."

Among the observations upon *Diseases* of *Bones* we find the following:—

"Bones fall more slowly into disease than the softer parts, and their restoration is proportionally more tardy; they receive their nutriment chiefly from the periosteum; and hence it is, that disease or injury to that membrane immediately affects the bone itself—a circumstance that should ever be borne in mind by the surgeon when operating upon bone; for it is scarcely possible that any very extensive destruction of periosteum can occur without exfoliation of the bone itself.

"The inflammation may be either acute or chronic, common or specific, and the strict antiphlogistic discipline, and counter-irritants, are the remedies to be adopted; with a severity to be regulated by the power of the patient's constitution. Calomel and opium will be found particularly useful when the pain is very urgent. It may be remarked, that the increase of pain at night is one of the diagnostic marks of the inflammation of bone, whether it be affected by simple or specific action; although it is too generally considered that the nocturnal pains indicate syphilitic affection, when, on the contrary, it seems to be concomitant with inflammation of bone from any cause. When the inflammatory symptoms have been relieved, or it has terminated by what is called resolution, a thickness of the inflamed bone remains, usually for a considerable time, from the difficulty there appears to be in the absorption of the adventitious earthy parts.

"When the inflammation goes on to the formation of matter, suppuration is indicated by symptoms similar to the formation of matter in soft parts, although there is somewhat more difficulty in deciding upon it, as no external marks at first present themselves; but rigors, with an increased sense of weight of the diseased part, and a remission of the severity of the pain, will usually lead to a just diagnosis. But in abscess, the part of the bone affected soon becomes swollen, its periosteum becomes thickened, caries supervenes, and the matter is discharged by a process similar to the discharge of matter from soft parts; but the progress and reparation are slower."

*Accidental Ossification* is thus described :—

"There are certain of the soft structures of the body, which are liable to accidental or unnatural depositions of bony matter, as the dura mater, pericardium, and even muscle. It generally happens, however, that the proportions of the animal and earthy parts are not the same as in the original bone. These deposits being sometimes as hard and polished as the enamel of the teeth, at other times soft and cretaceous, resembling moistened chalk. The former are most common in serous membranes, whilst the latter are not unfrequently met with in abscess of the lungs, in the uterus and ovaria."

*Application of the Trephine.*—"If there be a wound communicating with the bones of the skull, accompanied with fracture and depression, in such cases, although no untoward symptoms have as yet arisen, we are recommended immediately to trephine; but of the propriety of this step there seems to me some doubt. We should rather be led to judge of the necessity of the immediate application of the trephine by the *degree* of depression, and by the *part* of the skull injured; for as the quantity of diploe differs so much in different skulls, there is no evidence of the brain being injured until symptoms supervene; and I would therefore recommend that the patient should be most narrowly watched, and that the trephine should not be applied until symptoms point out the necessity. When compression arises from the formation of matter, which, as already mentioned, does not come on until some time after the accident, the surgeon is placed under the greatest difficulty to discover the precise seat of the injury, as it is not always at the part where the blow was inflicted. To ascertain this essential point, the scalp must be most carefully examined, and if a puffy appearance be found opposite to the part where the matter is situated, an incision is to be made through the part of the skull exposed, which will be found denuded, or at any rate its pericranium easily separable from it; the bone itself will be of an ash-colour, without any tendency to bleed. These circumstances will prove that you are justified in removing this portion of bone. I have seen my colleague, Mr. Key, under these circumstances, perform this operation with perfect success as far as relates to the evacuation of the matter, although the patient did not subsequently recover, in consequence of the extent of injury the brain had sustained."

The principal fractures are illustrated by lithographs.

